# Modification of lysine residues in proteins: a novel posttranslational effect of vitamin C

**DOI:** 10.1038/s41392-025-02288-7

**Published:** 2025-06-30

**Authors:** Dieter Kabelitz

**Affiliations:** Institute of Immunology, Christian-Albrechts University Kiel, Kiel, Germany

**Keywords:** Cell biology, Biochemistry

In a recent article published in *Cell*, He and colleagues reported that vitamin C (VitC) modifies lysine residues in proteins and peptides, thereby forming vitcyl-lysine, a process they have called vitcylation. They show that vitcylation of signal transducer and activator of transcription-1 (STAT1) increases its phosphorylation and thereby promotes interferon pathway activation in cancer cells and anti-tumor immunity.^1^

**Fig. 1:**
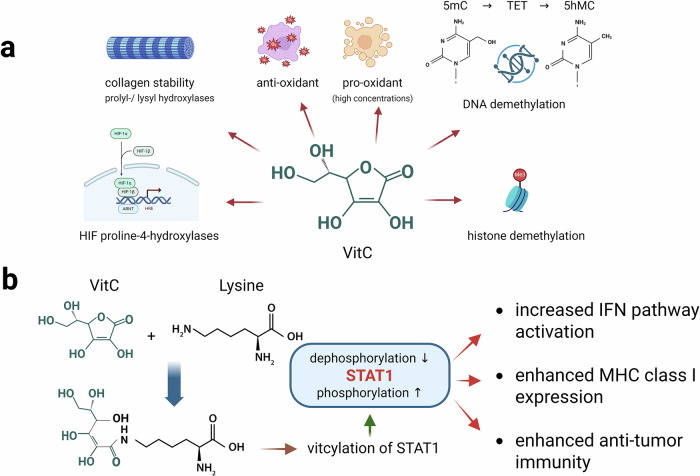
Molecular mechanisms of VitC action. **a** A summary of the well-described effects of VitC. VitC acts as a cofactor for numerous cytoplasmic enzymes, including dioxygenases. Examples are prolyl-/lysyl hydroxylases, which are required for correct folding of collagen fibers, or proline-4-hydroxylases involved in degradation of hypoxia-inducible factors (HIF), thereby regulating HIF-dependent gene expression. VitC is also a potent antioxidant, eliminating various reactive oxygen species (ROS), but can exert pro-oxidant activity at higher concentrations as well. Furthermore, VitC is an important epigenetic modifier. It acts as a cofactor for ten-eleven-translocation (TET) dioxygenases, which demethylate DNA by hydroxylating 5-methylcytosine (5mC) to 5-hydroxymethylcytosine (5hmC). Another level of epigenetic regulation is the methylation status of chromatin-deposited histones. VitC is an important cofactor for JmjC histone demethylases. **b** Vitcylation is a novel post-translational protein modification mediated by VitC. VitC modifies lysine residues in peptides and proteins. Vitcylation of signal transducer and activator of transcription (STAT)-1 enhances its phosphorylation by preventing dephosphorylation, thereby leading to increased interferon (IFN) pathway activation and upregulation of MHC class I expression in tumor cells associated with enhanced anti-tumor immunity. Figure created in https://BioRender.com

VitC, also known as L-ascorbic acid, has pleiotropic effects in cellular differentiation, signal transduction and immune cell regulation. Under physiological conditions, VitC occurs mainly as ascorbate anion, which can be oxidized to dehydroascorbic acid. VitC is a potent radical scavenger and antioxidant that efficiently detoxifies various reactive oxygen species.^1^ At higher concentrations, however, VitC also exerts pro-oxidant activity, which may induce oxidative stress and cell death in tumor cells.^2^ In support of its essential role in physiology, VitC acts as a cofactor for numerous enzymes, many of which are involved in crucial biochemical pathways. A major class of these enzymes comprises Fe^2+^-dependent dioxygenases, including collagen-modifying prolyl-/lysyl hydroxylases that are required for correct folding of collagen fibers and thus for stable formation of the extracellular matrix.^2^ VitC is also involved in the regulation of the activity of hypoxia-inducible factors (HIF) through the stimulation of proline hydroxylation.^2^ Moreover, VitC is an important epigenetic modifier acting at the level of DNA and histone demethylation. In this respect, VitC serves as a cofactor for ten-eleven-translocation (TET) enzymes, which remove cytosine methylation in DNA. Furthermore, VitC is also required for optimal catalytic activity of histone demethylases, which represents another crucial step of epigenetic regulation.^2,3^ Overall, the hitherto characterized modes of action of VitC encompass a broad spectrum of activities affecting diverse cellular systems in health and disease (Fig. 1a). Specifically, VitC has important functions in the regulation and differentiation of immune cells. Among many described effects, VitC enhances the differentiation of CD4 T cells from hematopoietic precursor cells, promotes the activation of regulatory T cells (Treg) through demethylation of the Treg-specific transcription factor *FOXP3*, and enhances the effector function of T cells, including γδ T cells.^4^

He and coworkers have now identified yet another molecular mechanism of action of VitC, i.e., the posttranslational modification of lysine residues in peptides and proteins by ascorbate anions. As noted by the authors, VitC contains a lactone structure that is also present in other substrates used for posttranslational modification of lysine residues in proteins (e.g., homocysteine thiolactone and succinic anhydride). To test whether VitC indeed modifies lysine residues in proteins, they added ascorbate anions to synthetic peptides containing lysine residues and performed matrix-assisted laser desorption/ionization–time-of-flight/time-of-flight mass spectrometry, which revealed a mass shift of the respective peptides, in line with VitC-induced peptide modification. Importantly, they demonstrated that the vitcylation was specific for lysine, and was abolished when lysine in the peptides was substituted with other amino acids such as cysteine, arginine, or alanine. In line with the sequence dependency of vitcylation, they found that not every lysine-containing peptide was modified. Further experiments with cell-free peptides revealed that vitcylation occurs in a concentration- and pH-dependent manner with an EC_50_ of 2 mM VitC and a permissive pH range of 7.0–9.5. Concentrations of VitC vary substantially across different tissues, but the doses of VitC required for lysine modification of proteins are well within the physiological range of intracellular VitC concentrations,^4^ suggesting that vitcylation may also take place in intact cells as well as in vivo. Consequently, the authors treated human and mouse cancer cell lines with 2 mM VitC and detected abundant vitcylated proteins in cells from both species with a significant overlap.

To further investigate the physiological significance of the newly identified protein modification, they performed expression analysis of VitC-treated murine cancer cells. Among the top-ranked genes in cancer cells exposed to VitC treatment were genes related to the interferon-response and inflammatory response pathway. A key factor in the interferon response is STAT1. In fact, the authors found that VitC treatment of human and mouse cancer cell lines induced vitcylation of a specific lysine residue (K298) in STAT1 associated with enhanced phosphorylation at tyrosine 701 and increased STAT1 activity. The analysis of various tumor cell lines revealed that vitcylation is a widely used mechanism of phosphorylation (and therefore activation) of STAT1 but not STAT2 nor STAT3. Mechanistically, the authors found that STAT1 vitcylation inhibited the interaction with the phosphatase TCPTP, thereby preventing the dephosphorylation of STAT1.

As a next step, the authors asked whether the enhanced STAT1 activation resulting from vitcylation and prolonged phosphorylation has any biological significance. Since STAT1 links the interferon pathway to antigen processing, they focused on the regulation of genes in the antigen processing pathway. Treatment of cancer cell lines with VitC upregulated various genes in the antigen processing pathway, as well as MHC class I cell surface expression. The authors excluded other mechanisms of VitC in this process such as antioxidant/pro-oxidant activity and TET enzyme activation, strongly arguing that STAT1 activation via vitcylation was the main mechanism. Next, they demonstrated that VitC-treated murine tumor cells expressing ovalbumin (OVA) elicited stronger responses of OVA-TCR transgenic CD8 T cells than untreated antigen-presenting cells. An elegant series of experiments indicated that enhanced antigen presentation was due to vitcylation of STAT1 and not due to other effects of VitC. Thus, VitC-induced effects were eliminated in STAT1-K298R mutant cells but not in TET2- or HIF-1α-deficient cells. Finally, the authors validated their in vitro results in immunocompetent mice transplanted with syngeneic tumor cells in the absence or presence of VitC treatment. Tumor growth was suppressed upon VitC treatment, and this effect could be again correlated with vitcylation of STAT1 in tumor cells, using similar strategies as in the in vitro experiments. Results of the study are summarized in Fig. 1b.

In this study, lysine modification by VitC has been discovered as a new posttranslational protein modification with potentially far-reaching implications for cell biology and cancer immunity. However, more work is required to fully appreciate the significance of vitcylation in relation to the other well-described effects of VitC, such as its role as a cofactor for enzymatic reactions and its antioxidant and pro-oxidant activity. While the authors noted a partial sequence dependency of lysine-specific protein modification, the range of proteins affected by this posttranslational modification needs to be investigated in more detail. STAT1 was identified as an important target for vitcylation, with implications for interferon pathway activation and anti-tumor immunity. High-dose VitC is known to enhance cancer immunotherapy, but the underlying mechanisms have so far not been clearly identified.^5^ The study by He et al. suggests that protein vitcylation might play a role in this scenario.^1^ It will be important in future studies to dissect the potential immuno-enhancing mechanisms of VitC at the level of posttranslational modification versus epigenetic regulation. As shown by the authors, a combination of VitC with other strategies, such as the application of checkpoint inhibitors, might further boost the anti-tumor immune response.
